# The national burden of influenza‐associated severe acute respiratory illness hospitalization in Zambia, 2011‐2014

**DOI:** 10.1111/irv.12492

**Published:** 2017-12-15

**Authors:** Andros Theo, Stefano Tempia, Adam L Cohen, Paul Simusika, Edward Chentulo, Chikama Mukwangole Chikamukwa, Mwaka Monze

**Affiliations:** ^1^ Cavendish University School of Medicine Lusaka Zambia; ^2^ Influenza Division Centers for Disease Control and Prevention Atlanta USA; ^3^ Influenza Program Centers for Disease Control and Prevention Pretoria South Africa; ^4^ Centre for Respiratory Diseases and Meningitis National Institute for Communicable Diseases of the National Health Laboratory Service Johannesburg South Africa; ^5^ Strategic Information Group Expanded Programme on Immunization Department of Immunization, Vaccines and Biological World Health Organization Geneva Switzerland; ^6^ National Influenza Center Virology Laboratory University Teaching Hospital Lusaka Zambia; ^7^ Lusaka Provincial Medical Office Ministry of Health Lusaka Zambia

**Keywords:** burden, hospitalization, influenza, severe acute respiratory illness, Zambia

## Abstract

**Background:**

Estimates of influenza‐associated hospitalization are limited in low‐ and middle‐income countries, especially in Africa.

**Objective:**

To estimate the national number of influenza‐associated severe acute respiratory illness (SARI) hospitalization in Zambia.

**Methods:**

We conducted active prospective hospital‐based surveillance for SARI at the University Teaching Hospital (UTH) situated in Lusaka Province during 2011‐2014. Upper respiratory tract samples were tested for influenza virus using a reverse transcriptase polymerase chain reaction assay. We estimated age‐specific rates of influenza‐associated SARI hospitalizations for the UTH using census and secondary data on respiratory hospitalizations following estimation approaches recommended by the World Health Organization. We used the UTH hospitalization rates as a proxy for Lusaka Province. These rates were adjusted for each of the remaining 9 provinces based on their prevalence of risk factors for pneumonia and healthcare‐seeking behavior. Rates were expressed per 100,000 population.

**Results:**

SARI cases accounted for 77.1% (13 389/17 354) of respiratory admissions at the UTH; 82.7% (11 859/14 344) and 50.8% (1530/3010) among individuals aged <5 and ≥5 years, respectively. Among SARI cases tested, the influenza virus detection rate was 5.5% (152/2734), 4.8% (48/998), and 6.0% (104/1736) among individuals aged <5 and ≥5 years, respectively. The mean annual national number of influenza‐associated SARI hospitalizations was 6181 (95% CI: 4321‐8041—rate: 43.9; 95% CI: 30.7‐57.1); 4669 (95% CI: 3287‐6051—rate: 187.7; 95% CI: 132.1‐243.3) among children aged <5 years; and 1512 (95% CI: 1037‐1987—rate: 13.1; 95% CI: 9.0‐17.2) among individuals aged ≥5 years.

**Conclusions:**

The burden of influenza‐associated SARI hospitalizations was substantial and was highest among children aged <5 years.

## 1 INTRODUCTION

Influenza virus infection is a major cause of severe acute respiratory illness (SARI) and results in significant global morbidity and mortality every year.^1^, ^2^, ^3^ However, the majority of available information on influenza disease burden emanates from industrialized countries. In recent years, influenza sentinel surveillance has been established in several African countries,^4^ and influenza virus infection has been reported to be associated with mild and severe illness including death on the Continent.^4^, ^5^ Nonetheless, estimates of the burden of influenza‐associated hospitalization are severely limited in Africa.

Zambia being part of the African Network for Influenza Surveillance and Epidemiology (ANISE) (http://www.cdc.gov/flu/international/anise.htm) is actively involved in surveillance of influenza‐associated illness mostly targeting patients with influenza‐like illnesses (ILI) and SARI at selected surveillance sites. This surveillance has provided a platform to monitor influenza virus circulation patterns and contributed to increase the understanding of the epidemiology of influenza viruses in the country.^6^

However, in Zambia, like in most countries in sub‐Saharan Africa, where influenza is often confused with febrile illnesses such as malaria and resource priorities are given to diseases such as HIV and tuberculosis,^7^ the impact of influenza‐associated severe illness on the general population remains poorly understood. The World Health Organization (WHO) highlighted that there is a need for influenza disease burden estimates especially from low‐ and middle‐income countries.^8^ These estimates would enable governments to make informed evidence‐based decisions when allocating scarce resources and planning intervention strategies to limit the impact and spread of the disease.

We aimed to estimate the national and provincial number and rates of SARI and influenza‐associated SARI hospitalizations among different age‐groups in Zambia from January 2011 through December 2014.

## 2 METHODS

### 2.1 Data sources

#### 2.1.1 Data source 1: Number of respiratory hospitalizations in Lusaka Province

We obtained the total number of respiratory hospitalizations for Lusaka Province by hospital from the Zambia Ministry of Health (ZMoH),^9^ which collects aggregated and de‐identified data on the number of admissions by syndrome from all public hospitals in Lusaka Province from January 2011 through December 2014.

#### 2.1.2 Data source 2: Number of any medical, respiratory, and SARI hospitalizations at the University Teaching Hospital

Over the same study period, we prospectively collected the total number of any medical and respiratory hospitalizations using hospital admission records at one large public hospital (the University Teaching Hospital—UTH) situated in Lusaka Province where influenza sentinel surveillance among patients hospitalized with SARI was implemented. The respiratory admissions were reclassified as SARI cases using clinical criteria consistent with the SARI case definition reported below.

#### 2.1.3 Data source 3: Influenza virus surveillance among patients hospitalized with SARI

We conducted prospective hospital‐based surveillance for SARI at the UTH from January 2011 through December 2014. The UTH was selected for the implementation of influenza sentinel surveillance because it is the largest referral hospital out of 9 hospitals serving the population of Lusaka Province and because of its proximity to the National Reference Laboratory for influenza. Surveillance activities were conducted at one pediatric and one adult inpatient wards where respiratory cases were admitted.

A case of SARI was defined as a hospitalized person who had illness onset within 7 days of admission and who met age‐specific clinical inclusion criteria. A case in children aged 2 days to <5 years included any hospitalized patient with cough or difficulty breathing and at least one of the following danger signs: unable to drink or breastfeed, lethargic, vomits everything, convulsion, chest indrawing, or stridor in a calm child. A case in persons aged ≥5 years included any hospitalized patient with fever (≥38°C), cough, and shortness of breath or difficulty breathing.^10^

The procedures of this surveillance program have been previously described.^6^ Briefly, trained surveillance nurses completed case report forms that included demographic, clinical, and epidemiological information for all enrolled SARI cases. In addition, respiratory specimens (nasopharyngeal and oropharyngeal swabs) were collected from all enrolled patients, placed in the same vial containing universal transport medium, stored at 4‐8°C and transported to the WHO‐accredited National Influenza Center (the UTH Virology Laboratory) within 72 hours of collection for testing. Specimens were tested for influenza A and B viruses using a real‐time reverse transcriptase polymerase chain reaction assay.^6^ Influenza A‐positive samples were further subtyped.^6^, ^11^

Verbal informed consent was obtained from all patients prior to data and specimen collection. For children <15 years, verbal consent was obtained from a parent or legal guardian.

#### 2.1.4 Data source 4: Prevalence of risk factors for pneumonia and healthcare‐seeking behavior for acute respiratory infection

We obtained the provincial‐level prevalence of known risk factors for pneumonia and the provincial data on healthcare‐seeking behavior among cases with acute respiratory infection (ARI) from the 2013‐2014 Zambia Demographic and Health Survey (DHS).^12^

#### 2.1.5 Data source 5: Population denominators

Provincial age‐ and year‐specific population denominators were obtained from projections of 2010 census data for Zambia.^13^ Zambia had a population of 14 676 147 individuals in 2014 of which 2 595 018 (17.7%) were children aged <5 years.

### 2.2 Estimation of the national number and rate of SARI and influenza‐associated SARI hospitalizations

**Figure 1:**
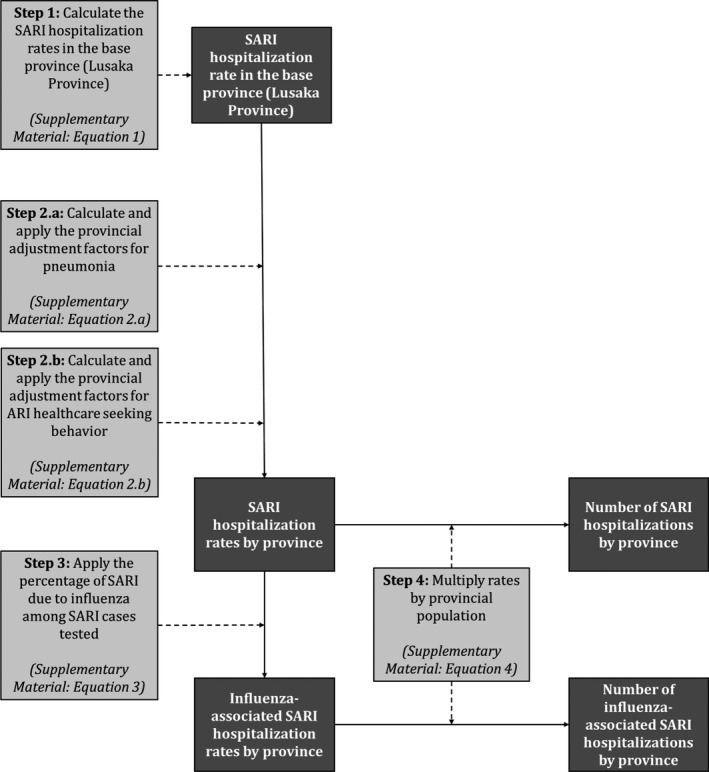
Method used to estimate the numbers and rates of severe acute respiratory illness (SARI) and influenza‐associated SARI hospitalizations in Zambia, 2011‐2014. Data inputs steps are in light gray boxes, and data outputs are in dark gray boxes

To estimate the national number and rates of SARI and influenza‐associated SARI hospitalization, we used a four‐step approach. In Step 1, we estimated the SARI hospitalizations rates in Lusaka Province (considered to be the base province in our estimation approach). In Step 2, we estimated the SARI hospitalizations rates for the other provinces from the base province using a previously described methodology.^14^, ^15^ In Step 3, we estimated the influenza‐associated SARI hospitalizations rates using available virological surveillance data for influenza. In Step 4, we obtained the number of SARI and influenza‐associated SARI hospitalizations using the estimated rates and the population at risk in each province. The description of the estimation approach for each step is provided below and in Figure 1. All estimates were obtained overall and within the following age categories: <1, 1‐4, 5‐24, 25‐44, 45‐64, ≥65, <5, and ≥5 years of age. Rates were expressed per 100,000 population. All estimates were reported as mean annual estimates over the study period.

#### 2.2.1 Step 1: Estimation of SARI hospitalization rates in Lusaka Province

To estimate the SARI hospitalization rates in Lusaka Province, we followed the WHO guidelines for estimating the disease burden associated with seasonal influenza.^8^ First, we estimated the service population of the UTH by dividing the number of respiratory hospitalizations at the UTH by the total number of respiratory hospitalizations in Lusaka Province using the hospitalization data obtained from the ZMoH (data source 1). This proportion was then applied to the population of Lusaka Province over the study period (data source 5). Thereafter, we obtained the SARI hospitalization rates for the UTH by dividing the total number of SARI hospitalizations at the UTH (data source 2) by the estimated service population. We used the UTH SARI hospitalization rates as a proxy for Lusaka Province as previously described.^14^, ^15^

#### 2.2.2 Step 2: Estimation of SARI hospitalizations rates in the other provinces

Estimates of SARI hospitalization rates for the other 9 provinces in Zambia were derived by adjusting the Lusaka Province rate (base province—obtained in Step 1) for the provincial‐level prevalence of known risk factors for pneumonia obtained from the DHS (data source 4) as previously described (Step 2.a).^14^, ^15^ Risk factors included HIV infection, exposure to indoor air pollution, crowding, malnutrition, low birthweight, and non‐exclusive breastfeeding.^14^, ^15^ The last three were only included for children aged <5 years. The relative risk of SARI associated with each risk factor was determined from the published literature.^14^, ^15^, ^16^, ^17^, ^18^ In addition, we adjusted the provincial rates by the proportion of ARI cases seeking care in the given province to the proportion of ARI cases seeking care in the base province using data from the DHS (data source 4) as previously described (Step 2.b).^14^, ^15^ The healthcare‐seeking behavior among ARI cases was used as a proxy for SARI cases. An adjustment factor >1 resulted in a greater SARI hospitalization rate in the given province relative to the base province and vice versa. The equations used for the provincial adjustments and the estimated adjustment factors (Table S1) are provided in the Supplementary Material.

#### 2.2.3 Step 3: Estimation of influenza‐associated SARI hospitalizations rates in all provinces

We estimated the provincial rates of influenza‐associated SARI hospitalization by multiplying the estimated provincial SARI hospitalization rates (obtained in Step 1 and 2) by the influenza virus detected rate obtained from influenza sentinel surveillance implemented among inpatients with SARI (data source 3).^14^, ^15^

#### 2.2.4 Step 4: Estimation of the number of SARI and influenza‐associated SARI hospitalizations in all provinces

We estimated the provincial number of SARI and influenza‐associated SARI hospitalizations by multiplying the provincial SARI (obtained in Steps 1 and 2) and influenza‐associated SARI (obtained in Step 3) hospitalization rates by the population at risk in each province (data source 5) over the study period.^14^, ^15^

We obtained the 95% confidence intervals (CI) using bootstrap resampling over 1000 replications for all parameters included in the calculations.^14^, ^15^ This included (i) the age‐ and year‐specific SARI hospitalization rates in the base province, (ii) the provincial prevalence of the risk factors for pneumonia, (iii) the provincial proportion of ARI cases seeking care, and (iv) the age‐specific influenza virus detection rate among SARI cases tested. The lower and upper limits of the 95% CI were the 2.5th and 97.5th percentiles of the estimated values obtained from the 1000 resampled datasets, respectively.

The statistical analysis was implemented using Stata 14.1 (StataCorp, College Station, Texas, USA).

### 2.3 Ethical approval

The influenza sentinel surveillance (data source 3) and the collection of aggregated data on any medical, respiratory, and SARI hospitalizations (data source 2) were deemed non‐research by the ZMoH and the US Centers for Disease Control and Prevention. The number of respiratory hospitalization in Lusaka Province (data source 1) and the DHS (data source 4) and census data (data source 5) were publicly available.

## 3 RESULTS

### 3.1 Number of hospitalizations in Lusaka Province and at the University Teaching Hospital

During 2011‐2014, the UTH accounted for 31.1% (15 678/50 388) of the respiratory hospitalizations reported for Lusaka Province to the ZMoH (data source 1). Over the same study period, 110 313 medical admissions were recorded from hospital record review (data source 2) at the UTH; 49 435 (44.8%) and 60 878 (55.2%) among patients aged <5 and ≥5 years, respectively. Of these, 17 354 (15.7%) were respiratory admissions; 14 344 (29.0%) among children aged <5 years, and 3010 (5.0%) among individuals aged ≥5 years. SARI cases accounted for 13 389 (77.1%) of respiratory hospitalizations; 11 859 (82.7%) among children aged <5 years, and 1530 (50.8%) among individuals aged ≥5 years. Children aged <5 years accounted for 88.6% (11 859/13 398) of total SARI hospitalizations.

### 3.2 Influenza virus surveillance among patients hospitalized with SARI

**Figure 2:**
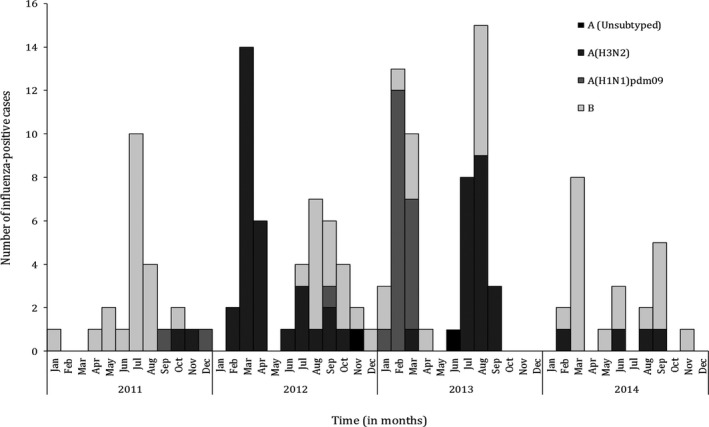
Monthly number of influenza‐positive cases among patients hospitalized with severe acute respiratory illness at the University Teaching Hospital, Lusaka Province, Zambia, 2011‐2014

During the study period, we enrolled and tested 2734 SARI cases at the UTH; 998 (36.5%) and 1736 (63.5%) cases among individuals aged <5 and ≥5 years, respectively (data source 3). Influenza viruses were detected in 152 (5.5%) specimens; 48 (4.8%) and 104 (6.0%) among individuals aged <5 and ≥5 years, respectively. Of these, 59 (38.7%) were influenza A(H3N2), 22 (14.6%) were influenza A(H1N1)pdm09, 2 (1.6%) were influenza A not subtyped, and 69 (45.2%) were influenza B viruses (Figure 2).

### 3.3 National number and rate of SARI and influenza‐associated SARI hospitalizations

The estimated mean annual number of SARI hospitalization was 118 668 (rate: 843.6 per 100 000 population); 95 223 (80.2%) (rate: 3828.4 per 100 000 population); and 23 444 (29.8%) (rate: 202.5 per 100 000 population) among individuals aged <5 and ≥5 years, respectively (Table 1). The estimated mean annual rates of SARI hospitalization were highest among children aged <1 year (11 548.3 per 100 000 population) and lowest among individuals aged 5‐24 years (95.2 per 100 000 population).

**Table 1:** Estimated mean annual numbers and rates of severe acute respiratory illness and influenza‐associated severe acute respiratory illness hospitalizations, Zambia, 2011‐2014

Age‐group (in years)	SARI hospitalizations		Influenza‐associated SARI hospitalizations	
	Number (95% CI)	Rate (95% CI)^a^	Number (95% CI)	Rate (95% CI)^a^
<1	57,449 (34,642‐80,256)	11,548.3 (6,963.6‐16,133)	2,494 (1,504‐3,484)	484.4 (292.3‐680.5)
1‐4	37,775 (22,136‐53,414)	1,898.4 (1,112.5‐2,684.3)	2,175 (1,275‐3,075)	109.3 (64.0‐154.6)
5‐24	6,525 (4,626‐8,424)	95.2 (67.5‐122.9)	406 (288‐524)	5.9 (4.2‐7.6)
25‐44	9,708 (6,737‐12,679)	297.8 (206.7‐388.9)	610 (423‐797)	18.7 (13‐24.4)
45‐64	4,211 (3,045‐5,377)	385.8 (278.9‐492.7)	283 (205‐361)	25.9 (18.7‐33.1)
≥65	3,000 (2,106‐3,894)	794.3 (557.6‐1,031.0)	214 (150‐278)	56.5 (39.7‐73.3)
<5	95,223 (67,037‐123,409)	3,828.4 (2,695.2‐4,961.6)	4,669 (3,287‐6,051)	187.7 (132.1‐243.3)
≥5	23,444 (16,083‐30,805)	202.5 (138.9‐266.1)	1,512 (1,037‐1,987)	13.1 (9.0‐17.2)
All	118,668 (82,948‐154,386)	843.6 (589.7‐1,097.5)	6,181 (4,321‐8,041)	43.9 (30.7‐57.1)
Province				
Central	10,148 (7,215‐13,081)	727.8 (517.5‐938.1)	529 (376‐682)	38.1 (27.3‐49.2)
Copperbelt	20,450 (13,988‐26,912)	981.6 (671.4‐1,291.8)	1,066 (729‐1,403)	51.2 (35.0‐67.4)
Eastern	15,328 (10,837‐19,819)	902.2 (637.9‐1,166.5)	800 (566‐1,034)	47.1 (33.3‐60.9)
Luapula	7,725 (5,098‐10,352)	731.9 (483.1‐980.7)	402 (265‐539)	38.1 (25.1‐51.1)
Lusaka	21,843 (15,421‐28,265)	889.7 (628.1‐1,151.3)	1,132 (799‐1,465)	46.1 (32.5‐59.7)
Muchinga	5,949 (4,123‐7,775)	774 (536.4‐1,011.6)	310 (215‐405)	40.4 (28.0‐52.8)
North Western	7,439 (5,334‐9,544)	968.7 (694.6‐1,242.8)	388 (278‐498)	50.6 (36.3‐64.9)
Northern	8,296 (5,674‐10,918)	692.9 (473.9‐911.9)	432 (295‐569)	36.1 (24.7‐47.5)
Southern	13,709 (9,624‐17,794)	804.3 (564.6‐1,044)	715 (502‐928)	42.0 (29.5‐54.5)
Western	7,781 (5,602‐9,960)	826 (594.7‐1,057.3)	407 (293‐521)	43.2 (31.1‐55.3)

SARI, severe acute respiratory illness; CI, confidence intervals.

a Rates expressed per 100,000 population.

The estimated mean annual number of influenza‐associated SARI hospitalization was 6181 (rate: 43.2 per 100 000 population); 4669 (75.5%) (rate: 187.7 per 100 000 population); and 1521 (24.5%) (rate: 13.1 per 100 000 population) among individuals aged <5 and ≥5 years, respectively (Table 1). The estimated mean annual rates of influenza‐associated SARI hospitalization were highest among children aged <1 year (484.4 per 100 000 population) and lowest among individuals aged 5‐24 years (5.9 per 100 000 population).

A U‐shaped trend of the magnitude of the SARI and influenza‐associated SARI hospitalizations rates was observed across age‐groups (Table 1). No significant difference (overlapping CIs) of the SARI and influenza‐associated SARI hospitalizations rates was observed across Provinces (Table 1). The provincial number and rates of SARI and influenza‐associated SARI hospitalizations by age‐group are provided in Table S2.

## 4 DISCUSSION

We provide estimates of the national burden of influenza‐associated SARI hospitalization in Zambia over a 4‐year period. The results indicate a sizeable number of influenza‐associated SARI hospitalizations across age‐groups. However, higher rates of influenza‐associated SARI hospitalizations occurred among children aged <5 years and persons aged ≥65 years. Approximately three‐quarters of the total number of influenza‐associated SARI hospitalizations occurred in children aged <5 years.

A global study on influenza burden reported influenza‐associated respiratory hospitalizations rates of 174 per 100 000 population among African children aged <5 years.^3^ Other Africa studies conducted using population‐based surveillance among children aged <5 years estimated rates of influenza‐associated SARI hospitalization of 135 per 100 000 population in Ghana,^19^ 270 per 100 000 population in Kenya,^20^ and 153‐186 per 100 000 population in South Africa.^21^ Our finding of influenza‐associated hospitalization rates in children <5 years (187 per 100 000 population) is generally consistent with the estimates obtained from similar studies conducted in Africa, albeit higher compared to those of other regions.^3^

In our study, compared to children aged <5 years, the burden of influenza‐associated SARI hospitalizations was lower in individuals aged ≥5 years (13 per 100 000 population). This was observed also in other studies conducted in Africa.^19^, ^20^, ^21^ Our estimates of influenza‐associated SARI hospitalization in individuals aged ≥5 years are on the lower end, but overall similar to other estimates from Africa (22 per 100 000 population in South Africa 21 and 30 per 100 000 population in Kenya 20). Underlying medical conditions including HIV infection is known risk factors for influenza‐associated severe illness.^21^, ^22^, ^23^, ^24^ Differences in the prevalence of such conditions in different settings may explain some of the observed differences in the influenza‐associated SARI hospitalization rates in this age‐group. Cultural differences and differential access to health care across different countries can also play a role in differential healthcare‐seeking behavior that in return may be responsible for differences in hospitalization rates.

In our study, we did not observe significant differences in the provincial rates of influenza‐associated SARI hospitalizations. This was observed also in studies conducted in Kenya 14 and South Africa.^15^ This may suggest that geographical variations within countries may not significantly affect influenza disease burden estimates.

In our study, the age‐specific SARI hospitalization rates were higher among children aged <5 years (3828.4 per 100 000 population) and declined among individuals aged ≥5 (202.5 per 100 000 population). SARI hospitalization rates of 2530‐3173 per 100 000 population 18 and 325‐389 per 100 000 17 have been reported in South Africa among children aged <5 years and individuals aged ≥5 years, respectively.

No well‐defined seasonal patterns on influenza virus circulation were observed, and this was consisted with findings from other tropical African countries.^4^

Our study has limitations that warrant discussion. First, whereas we estimated national numbers and rates of SARI and influenza‐associated SARI hospitalizations using a previously described methodology,^14^, ^15^ our estimates were derived from SARI surveillance conducted at one sentinel hospital used as a proxy to obtain rates from the base Province. The influenza virus detection rate may vary in different locations in Zambia; however, this could not be investigated. Nonetheless, the influenza virus detection rate among SARI cases was found to be similar across 15 African countries including Zambia (median: 8.9%; interquartile range: 5.7%‐11.6%),^4^ and our rates of SARI and influenza‐associated SARI hospitalizations were similar to those obtained from population‐based surveillance in other African countries.^19^, ^20^, ^21^ In addition, we used bootstrapping for the calculation of the CIs to account for the level of uncertainty associated with all adjustments used in our estimation approach as previously reported.^14^, ^15^ Second, whereas we prospectively collected the total number of SARI cases across UTH wards, the admission ward was not recorded in this dataset, hindering our ability to estimate the proportion of underenrollment at the wards where surveillance was implemented. Third, we were unable to account for patients hospitalized for respiratory illness that did not meet the SARI case definition. Influenza virus infection has been reported also among patients hospitalized for respiratory illness that do not meet the SARI case definition. Specifically in a study conducted in South Africa, influenza virus was detected in 5.8% of patients hospitalized with respiratory illnesses that did not meet the SARI case definition.^25^ Last, ecological studies have suggested that influenza virus is responsible for hospitalizations and deaths also among patients presenting with circulatory illnesses or even syndromes different than respiratory and circulatory.^23^, ^26^ In addition, individuals that may have developed influenza‐associated severe respiratory illness, but did not seek care, would have been missed in our study; hence, our estimates should be considered minimum estimates.

In conclusion, we estimated that there were between 4312 and 8041 influenza‐associated SARI hospitalizations each year in Zambia. The hospitalization rates were elevated in children aged <5 years and individuals aged ≥65 years. The ZMoH is yet to implement a national influenza vaccination program. Should an influenza vaccination program be introduced in Zambia, young children and the elderly may benefit most from annual influenza immunization. No influenza vaccine is licensed for children aged <6 months, but this group may be protected through the vaccination of their mothers during pregnancy.^27^, ^28^ However, given the limited financial resources in our setting, estimation of the disease burden associated with other pathogens should also be considered to inform prioritization of interventions.

## DISCLAIMER

The findings and conclusions in this report are those of the authors and do not necessarily represent the official position of the US Centers for Disease Control and Prevention, USA, or the Zambia Ministry of Health.

## CONFLICT OF INTEREST

All authors declare that they have no commercial or other associations that may pose a conflict of interest.

## ETHICS

The influenza sentinel surveillance and the collection of aggregated data on any medical, respiratory, and SARI hospitalizations were deemed non‐research by the ZMoH and the US Centers for Disease Control and Prevention. The number of respiratory hospitalization in Lusaka Province and the DHS and census data were publicly available.

## AUTHOR CONTRIBUTIONS

All authors take responsibility for the integrity of the data and the accuracy of the data analysis. Andros Theo, Stefano Tempia, Adam Cohen, and Paul Simusika performed study concept and design. Andros Theo, Stefano Tempia, Adam Cohen, Paul Simusika, Edward Chentulo, Chikama Mukwangole Chikamukwa, and Mwaka Monze involved in the acquisition, analysis, or interpretation of data. Andros Theo, Stefano Tempia, Adam Cohen, and Paul Simusika drafted the manuscript. Andros Theo, Stefano Tempia, Adam Cohen, Paul Simusika, Edward Chentulo, Chikama Mukwangole Chikamukwa, and Mwaka Monze involved in the critical revision of the manuscript for important intellectual content.
