# Supplementary material for: The national burden of influenza‐associated severe acute respiratory illness hospitalization in Zambia, 2011‐2014
Source: Influenza Other Respir Viruses. 2017 Dec 15;12(1):46–53. doi: 10.1111/irv.12492 (PMC5818337; doi:10.1111/irv.12492)
Supplement: Supplementary file 1 [file IRV-12-46-s001.docx]

**The National Burden of Influenza-Associated Severe Acute Respiratory Illness Hospitalization in Zambia, 2011-2014 (Supplementary Material)**

**Authors**

Andros Theo^1^, Stefano Tempia^2,3,4^, Adam L Cohen^1,5^, Paul Simusika^6^, Edward Chentulo^6^, Chikama Mukwangole Chikamukwa^7^, Mwaka Monze^6^

**Affiliations**

^1^Cavendish University School of Medicine, Lusaka, Zambia.

^2^ Influenza Division, Centers for Disease Control and Prevention, Atlanta, United States of America

^3^ Influenza Program, Centers for Disease Control and Prevention, Pretoria, South Africa.

^4^ Centre for Respiratory Diseases and Meningitis, National Institute for Communicable Diseases of the National Health Laboratory Service, Johannesburg, South Africa.

^5^ Strategic Information Group, Expanded Programme on Immunization, Department of Immunization, Vaccines and Biological, World Health Organization, Geneva, Switzerland.

^6^ National Influenza Center, Virology Laboratory, University Teaching Hospital, Lusaka, Zambia.

^7^Lusaka Provincial Medical Office, Ministry of Health, Longacres, Lusaka, Zambia.

**METHODS**

**Equations used to estimate the provincial numbers and rates of SARI and influenza-associated SARI hospitalizations**

***Equation 1:*** *SARI hospitalization rates in the base province (Lusaka Province)*

$${RS}_{H,B}=\frac{{SARI}_{H,B}}{{Pop}_{B}}$$

Where:

${RS}_{H,B}$ = Base rate of hospitalized SARI

${SARI}_{H,B}$ = Number of total cases meeting SARI case definition hospitalized at the University Teaching Hospital (UTH) (Lusaka Province)

${Pop}_{B}$ = Population of UTH catchment area

***Equation 2.a:*** *Calculation of adjustment for risk factors at the provincial level for base SARI hospitalization rates*

$${Adj}_{Y}=\left( 1+\sum_{i} \left( P_{i,Y}-P_{i,B} \right)\times\left( {RR}_{i}-1 \right) \right)$$

Where:

${Adj}_{Y}$ = Adjustment factor for province Y for risk factors of SARI

$P_{i,Y}$ = Prevalence of risk factor *i* in province Y

$P_{i,B}$= Prevalence of risk factor *i* in base province

${RR}_{i}$ = Relative risk of SARI due to risk factor *i*

***Equation 2.b:*** *SARI hospitalization rates in the given province after adjustment for risk factors and healthcare-seeking behavior*

$${RS}_{H,Y}={RS}_{H,B} \times{Adj}_{Y} \times\frac{{DHS}_{H,Y}}{{DHS}_{H,B}}$$

Where:

${RS}_{H,Y}$ = Rate of hospitalized SARI in province Y

${DHS}_{H,Y}$ = Proportion of ARI cases seeking care in province Y (from DHS)

${DHS}_{H,B}$ = Proportion of ARI cases seeking care in base province (from DHS)

***Equation 3:*** *Influenza-associated SARI hospitalization rates in all province*

$${RI}_{H,Y}={RS}_{H,Y} \times I$$

Where:

${RI}_{H,Y}$= Rate of hospitalized influenza-associated SARI in province Y

(including base province)

$I$ = Proportion of hospitalized SARI cases testing positive for influenza

***Equation 4:*** *Number of SARI and influenza-associated SARI hospitalizations in all provinces*

$${NS}_{H,Y}={RS}_{H,Y} \times P{op}_{Y}$$

Where:

${NS}_{H,Y}$= Number of hospitalized SARI cases in province Y (including base

province)

$P{op}_{Y}$= Population in province Y (including base province)

$${NI}_{H,Y}={RI}_{H,Y} \times P{op}_{Y}$$

Where:

${NI}_{H,Y}$= Number of hospitalized influenza-associated SARI cases in province Y

(including base province)

$P{op}_{Y}$= Population in province Y (including base province)

**RESULTS**

**Table S1: Adjustment factors for provincial estimates of severe acute respiratory illness hospitalization rates, Zambia, 2011-2014**

| **Age group (in years)** | **Adjustment factors** | | |
| --- | --- | --- | --- |
|  | **Risk factors for pneumonia** | **ARI healthcare seeking behavior** | **Combined** |
| **Lusaka (Base province)** | | | |
| <5 | 1 | 1 | 1 |
| ≥5 | 1 | 1 | 1 |
| **Central** | | | |
| <5 | 1.0304 | 0.9188 | 0.9469 |
| ≥5 | 1.0004 | 0.9188 | 0.9192 |
| **Copperbelt** | | | |
| <5 | 1.0721 | 1.1914 | 1.2773 |
| ≥5 | 1.0392 | 1.1914 | 1.2381 |
| **Eastern** | | | |
| <5 | 0.9920 | 1.1792 | 1.1697 |
| ≥5 | 0.9788 | 1.1792 | 1.1542 |
| **Luapula** | | | |
| <5 | 1.0921 | 0.8790 | 0.9600 |
| ≥5 | 1.0168 | 0.8790 | 0.8938 |
| **Machinga** | | | |
| <5 | 1.0637 | 0.9479 | 1.0083 |
| ≥5 | 1.0252 | 0.9479 | 0.9718 |
| **North Western** | | | |
| <5 | 1.0351 | 1.2129 | 1.2554 |
| ≥5 | 1.0236 | 1.2129 | 1.2415 |
| **Northern** | | | |
| <5 | 1.0281 | 0.8836 | 0.9084 |
| ≥5 | 0.9596 | 0.8836 | 0.8479 |
| **Southern** | | | |
| <5 | 1.0021 | 1.0429 | 1.0451 |
| ≥5 | 0.9784 | 1.0429 | 1.0204 |
| **Western** | | | |
| <5 | 0.9960 | 1.0704 | 1.0662 |
| ≥5 | 1.0044 | 1.0704 | 1.0752 |

**Table S2: Estimated mean annual numbers and rates of severe acute respiratory illness and influenza-associated severe acute respiratory illness hospitalizations by age group and province, Zambia, 2011-2014.**

| **Age group (in years)** | **SARI hospitalizations** | | **Influenza-associated SARI hospitalizations** | |
| --- | --- | --- | --- | --- |
|  | **Number (95% CI)** | **Rate (95% CI)^a^** | **Number (95% CI)** | **Rate (95% CI) ^a^** |
| **Central** | | | | |
| <1 | 4,888 (2,845-6,931) | 9,913.9 (5,769.9-14,057.9) | 212 (123-301) | 430.4 (250.5-610.3) |
| 1-4 | 3,214 (1,928-4,500) | 1,629.7 (977.8-2,281.6) | 185 (111-259) | 93.8 (56.3-131.3) |
| 5-24 | 569 (401-737) | 83.9 (59.1-108.7) | 35 (25-45) | 5.2 (3.7-6.7) |
| 25-44 | 847 (587-1,107) | 262.2 (181.7-342.7) | 53 (37-69) | 16.5 (11.4-21.6) |
| 45-64 | 367 (252-482) | 339.6 (233.6-445.6) | 25 (17-33) | 22.8 (15.7-29.9) |
| ≥65 | 262 (181-343) | 699.3 (483.9-914.7) | 19 (13-25) | 49.8 (34.5-65.1) |
| <5 | 8,103 (5,794-10,412) | 3,286.5 (2,349.8-4,223.2) | 397 (284-510) | 161.1 (115.2-207.0) |
| ≥5 | 2,046 (1,408-2,684) | 178.2 (122.6-233.8) | 132 (91-173) | 11.5 (7.9-15.1) |
| All | 10,148 (7,215-13,081) | 727.8 (517.5-938.1) | 529 (376-682) | 38.1 (27.3-49.2) |
| **Copperbelt** | | | | |
| <1 | 9,854 (6,031-13,677) | 13,375.3 (8,185.7-18,564.9) | 428 (262-594) | 580.7 (355.4-806.0) |
| 1-4 | 6,479 (3,972-8,986) | 2,198.7 (1,347.8-3,049.6) | 373 (229-517) | 126.6 (77.6-175.6) |
| 5-24 | 1,146 (815-1,477) | 112.9 (80.3-145.5) | 71 (50-92) | 7.3 (5.6-9.1) |
| 25-44 | 1,705 (1,231-2,179) | 353.2 (255-451.4) | 107 (77-137) | 22.2 (16.7-28.4) |
| 45-64 | 739 (530-948) | 457.5 (328-587) | 50 (36-64) | 30.8 (22.1-39.5) |
| ≥65 | 527 (376-678) | 941.9 (672.5-1,211.3) | 38 (27-49) | 67.1 (47.9-86.3) |
| <5 | 16,333 (11,253-21,413) | 4,434.7 (3,055.3-5,813.9) | 801 (552-1,050) | 217.4 (149.8-285.6) |
| ≥5 | 4,117 (2,919-5,315) | 240.1 (170.2-310) | 266 (189-343) | 15.5 (11.2-20.3) |
| All | 20,450 (13,988-26,912) | 981.6 (671.4-1,291.8) | 1,066 (729-1,403) | 51.2 (35.0-67.4) |
| **Eastern** | | | | |
| <1 | 7,359 (4,504-10,214) | 12,248.7 (7,496.2-17,001.2) | 319 (195-443) | 531.8 (325.5-738.1) |
| 1-4 | 4,839 (2,942-6,736) | 2,013.5 (1,224.2-2,802.8) | 279 (170-388) | 115.9 (70.5-161.3) |
| 5-24 | 871 (597-1,145) | 105.3 (72.1-138.5) | 54 (37-71) | 6.5 (4.5-8.5) |
| 25-44 | 1,296 (916-1,676) | 329.2 (232.7-425.7) | 81 (57-105) | 20.7 (14.6-26.8) |
| 45-64 | 562 (397-727) | 426.5 (301.5-551.5) | 38 (27-49) | 28.7 (20.3-37.1) |
| ≥65 | 401 (277-525) | 878.0 (607.6-1,148.4) | 29 (20-38) | 62.5 (43.3-81.7) |
| <5 | 12,198 (8,514-15,882) | 4,060.5 (2,834.2-5,286.8) | 598 (417-779) | 199.1 (139.0-259.2) |
| ≥5 | 3,130 (2,178-4,082) | 223.8 (155.8-291.8) | 202 (141-263) | 14.4 (10.0-18.8) |
| All | 15,328 (10,837-19,819) | 902.2 (637.9-1,166.5) | 800 (566-1,034) | 47.1 (33.3-60.9) |
| **Luapula** | | | | |
| <1 | 3,752 (2,262-5,242) | 10,052.2 (6,061.5-14,042.9) | 163 (98-228) | 436.4 (263.1-609.7) |
| 1-4 | 2,467 (1,443-3,491) | 1,652.4 (966.7-2,338.1) | 142 (83-201) | 95.1 (55.6-134.6) |
| 5-24 | 419 (288-550) | 81.5 (56.1-106.9) | 26 (18-34) | 5.1 (3.5-6.7) |
| 25-44 | 624 (441-807) | 255.0 (180.3-329.7) | 39 (28-50) | 16 (11.3-20.7) |
| 45-64 | 270 (192-348) | 330.2 (235.1-425.3) | 18 (13-23) | 22.2 (15.8-28.6) |
| ≥65 | 193 (136-250) | 679.9 (478-881.8) | 14 (10-18) | 48.4 (34.0-62.8) |
| <5 | 6,219 (4,272-8,166) | 3,332.4 (2,289.4-4,375.4) | 305 (210-400) | 163.4 (112.3-214.5) |
| ≥5 | 1,506 (1,051-1,961) | 173.3 (121.0-225.6) | 97 (68-126) | 11.2 (7.8-14.6) |
| All | 7,725 (5,098-10,352) | 731.9 (483.1-980.7) | 402 (265-539) | 38.1 (25.1-51.1) |
| **Lusaka** | | | | |
| <1 | 10,814 (6,542-15,086) | 12,455.4 (7,535.5-17,375.3) | 469 (284-654) | 540.7 (327.1-754.3) |
| 1-4 | 7,110 (4,202-10,018) | 2,047.5 (1,210.1-2,884.9) | 409 (242-576) | 117.9 (69.7-166.1) |
| 5-24 | 1,091 (766-1,416) | 91.2 (64.0-118.4) | 68 (48-88) | 5.7 (4.0-7.4) |
| 25-44 | 1,623 (1,134-2,112) | 285.3 (199.4-371.2) | 102 (71-133) | 17.9 (12.5-23.3) |
| 45-64 | 704 (486-922) | 369.5 (255.6-484.8) | 47 (32-62) | 24.8 (17.1-32.5) |
| ≥65 | 501 (354-648) | 760.7 (537.8-983.6) | 36 (25-47) | 54.2 (38.3-70.1) |
| <5 | 17,924 (13,085-22,763) | 4,129.0 (3,014.2-5,243.8) | 879 (642-1,116) | 202.5 (147.8-257.2) |
| ≥5 | 3,919 (2,849-4,989) | 193.9 (141-246.8) | 253 (184-322) | 12.5 (9.1-15.9) |
| All | 21,843 (15,421-28,265) | 889.7 (628.1-1,151.3) | 1,132 (799-1,465) | 46.1 (32.5-59.7) |
| **Muchinga** | | | | |
| <1 | 2,870 (1,742-3,998) | 10,558.4 (6,408.9-14,707.9) | 125 (76-174) | 458.4 (278.2-638.6) |
| 1-4 | 1,887 (1,166-2,608) | 1,735.6 (1,072.6-2,398.6) | 109 (67-151) | 99.9 (61.7-138.1) |
| 5-24 | 332 (224-440) | 88.7 (60.0-117.4) | 21 (14-28) | 5.5 (3.7-7.3) |
| 25-44 | 494 (361-627) | 277.2 (202.6-351.8) | 31 (23-39) | 17.4 (12.7-22.1) |
| 45-64 | 214 (151-277) | 359.1 (252.8-465.4) | 14 (10-18) | 24.1 (17.0-31.2) |
| ≥65 | 153 (103-203) | 739.3 (499.8-978.8) | 11 (7-15) | 52.6 (35.6-69.6) |
| <5 | 4,757 (3,216-6,298) | 3,500.2 (2,366.1-4,634.3) | 233 (158-308) | 171.6 (116.0-227.2) |
| ≥5 | 1,192 (833-1,551) | 188.4 (131.7-245.1) | 77 (54-100) | 12.2 (8.5-15.9) |
| All | 5,949 (4,123-7,775) | 774 (536.4-1,011.6) | 310 (215-405) | 40.4 (28.0-52.8) |
| **North Western** | | | | |
| <1 | 3,570 (2,124-5,016) | 13,146.1 (7,821.9-18,470.3) | 155 (92-218) | 570.7 (339.6-801.8) |
| 1-4 | 2,347 (1,368-3,326) | 2,161 (1,259.9-3,062.1) | 135 (79-191) | 124.4 (72.5-176.3) |
| 5-24 | 424 (302-546) | 113.2 (80.7-145.7) | 26 (19-33) | 7.4 (5.6-9.5) |
| 25-44 | 630 (447-813) | 354.2 (251.5-456.9) | 40 (28-52) | 22.2 (15.8-28.6) |
| 45-64 | 273 (193-353) | 458.7 (323.8-593.6) | 18 (13-23) | 30.8 (21.7-39.9) |
| ≥65 | 195 (139-251) | 944.4 (672.4-1,216.4) | 14 (10-18) | 67.2 (47.8-86.6) |
| <5 | 5,917 (4,160-7,674) | 4,358 (3,063.7-5,652.3) | 290 (204-376) | 213.7 (150.2-277.2) |
| ≥5 | 1,522 (1,100-1,944) | 240.7 (174-307.4) | 98 (71-125) | 15.5 (11.2-19.8) |
| All | 7,439 (5,334-9,544) | 968.7 (694.6-1,242.8) | 388 (278-498) | 50.6 (36.3-64.9) |
| **Northern** | | | | |
| <1 | 4,027 (2,452-5,602) | 9,512.6 (5,793.2-13,232) | 175 (107-243) | 413.0 (251.5-574.5) |
| 1-4 | 2,648 (1,560-3,736) | 1,563.7 (921-2,206.4) | 152 (90-214) | 90.1 (53.8-127.6) |
| 5-24 | 451 (319-583) | 77.3 (54.7-99.9) | 28 (20-36) | 4.8 (3.4-6.2) |
| 25-44 | 671 (476-866) | 241.9 (171.5-312.3) | 42 (30-54) | 15.2 (10.8-19.6) |
| 45-64 | 291 (206-376) | 313.3 (221.5-405.1) | 20 (14-26) | 21.1 (14.9-27.3) |
| ≥65 | 207 (143-271) | 645 (444.4-845.6) | 15 (10-20) | 45.9 (31.6-60.2) |
| <5 | 6,675 (4,586-8,764) | 3,153.5 (2,166.5-4,140.5) | 327 (225-429) | 154.6 (106.2-203.0) |
| ≥5 | 1,620 (1,105-2,135) | 164.4 (112.1-216.7) | 105 (72-138) | 10.6 (7.2-14) |
| All | 8,296 (5,674-10,918) | 692.9 (473.9-911.9) | 432 (295-569) | 36.1 (24.7-47.5) |
| **Southern** | | | | |
| <1 | 6,596 (4,109-9,083) | 10,943.3 (6,817.7-15,068.9) | 286 (178-394) | 475.1 (296.0-654.2) |
| 1-4 | 4,337 (2,611-6,063) | 1,798.9 (1,082.9-2,514.9) | 250 (150-350) | 103.6 (62.4-144.8) |
| 5-24 | 773 (550-996) | 93.1 (66.2-120) | 48 (34-62) | 5.8 (4.1-7.5) |
| 25-44 | 1,149 (823-1,475) | 291.1 (208.4-373.8) | 72 (52-92) | 18.3 (13.1-23.5) |
| 45-64 | 499 (346-652) | 377 (261.6-492.4) | 34 (24-44) | 25.4 (17.6-33.2) |
| ≥65 | 355 (244-466) | 776.2 (534-1,018.4) | 25 (17-33) | 55.3 (38.0-72.6) |
| <5 | 10,933 (7,675-14,191) | 3,627.8 (2,546.7-4,708.9) | 536 (376-696) | 177.9 (124.9-230.9) |
| ≥5 | 2,776 (1,960-3,592) | 197.9 (139.7-256.1) | 179 (126-232) | 12.8 (9.0-16.6) |
| All | 13,709 (9,624-17,794) | 804.3 (564.6-1,044) | 715 (502-928) | 42.0 (29.5-54.5) |
| **Western** | | | | |
| <1 | 3,719 (2,246-5,192) | 11,164.1 (6,743.1-15,585.1) | 161 (97-225) | 484.7 (292.8-676.6) |
| 1-4 | 2,445 (1,472-3,418) | 1,835.2 (1,104.8-2,565.6) | 141 (85-197) | 105.7 (63.6-147.8) |
| 5-24 | 450 (324-576) | 98.1 (70.5-125.7) | 28 (20-36) | 6.1 (4.4-7.8) |
| 25-44 | 669 (466-872) | 306.7 (213.8-399.6) | 42 (29-55) | 19.3 (13.5-25.1) |
| 45-64 | 290 (205-375) | 397.3 (280.5-514.1) | 20 (14-26) | 26.7 (18.9-34.5) |
| ≥65 | 207 (144-270) | 817.9 (570.1-1065.7) | 15 (10-20) | 58.2 (40.6-75.8) |
| <5 | 6,165 (4,297-8,033) | 3,701.0 (2,579.6-4,822.4) | 302 (210-394) | 181.5 (126.5-236.5) |
| ≥5 | 1,617 (1,148-2,086) | 208.5 (148-269) | 104 (74-134) | 13.4 (9.5-17.3) |
| All | 7,781 (5,602-9,960) | 826 (594.7-1,057.3) | 407 (293-521) | 43.2 (31.1-55.3) |

Abbreviations: SARI: severe acute respiratory illness; CI: confidence intervals.

^a^ Rates expressed per 100,000 population.
